# Macrophage polarization is involved in liver fibrosis induced by β _1_-adrenoceptor autoantibody: β _1_-Adrenoceptor autoantibody and liver fibrosis

**DOI:** 10.3724/abbs.2022102

**Published:** 2022-08-16

**Authors:** Ye Wu, Xiongxiong Fan, Haicun Yu, Jingyi Liu, Yanru Duan, Suli Zhang, Li Yan, Yunhui Du, Huirong Liu

**Affiliations:** 1 Department of Physiology and Pathophysiology School of Basic Medical Sciences Capital Medical University Beijing 100069 China; 2 Beijing key laboratory of metabolic disorder related cardiovascular disease School of Basic Medical Sciences Capital Medical University Beijing 100069 China; 3 Zhengzhou Central Hospital affiliated of Zhengzhou University Zhengzhou 450000 China; 4 Shanxi Cardiovascular Hospital Cardiac Care Unit Taiyuan 030024 China; 5 Department of Pathophysiology Institute of Basic Medical Science Chinese Academy of Medical Sciences & Peking Union Medical College Beijing 100005 China; 6 Beijing Anzhen Hospital Capital Medical University Beijing Institute of Heart Lung and Blood Vessel Diseases Beijing100029 China

**Keywords:** β _1_-adrenergic receptor, autoantibody, hepatic fibrosis, macrophages

## Abstract

Accumulating evidence suggests that liver injury can be induced by the over-expression of β _1_-adrenergic receptors (β _1_-ARs). High titers of autoantibodies specific to β _1_-adrenergic receptors (β _1_-AA) are detected in the sera of heart failure patients, potentially playing agonist-like roles. However, the role of β _1_-AA in liver function has not been characterized. In this study, we collect the sera of primary biliary cholangitis (PBC) patients, a condition which easily develops into liver fibrosis, and analyze the relationship between PBC and β _1_-AA. A passive immunization model is established to assess the effect of β _1_-AA on the liver. Subsequently, the effect of β _1_-AA on macrophages is investigated *in vitro*. Results show that PBC patients have a high titer and ratio of β _1_-AA, compared to controls. Liver injury and fibrosis are induced by β _1_-AA. *In vitro* experiments with ROS probe demonstrate that β _1_-AA induces macrophages to produce ROS and secrete TNFα. These effects can be partially reversed by metoprolol, a blocker for β _1_-AR. Results from the transwell and phagocytosis assays show that β _1_-AA promotes macrophage migration and phagocytosis. FCM tests suggest that β _1_-AA induces the alteration of M1 rather than M2 markers in macrophages. Finally, the Annexin V/PI assay indicates that macrophage culture supernatants stimulated by β _1_-AA cause hepatocyte apoptosis. Overall, these results suggest that β _1_-AA is involved in PBC. The β _1_-AA-induced activation, phagocytosis and phenotypic modification of macrophages may play an important role in the development of hepatic fibrosis and injury.

## Introduction

Recent research has highlighted that organ fibrosis causes 45% of total deaths per year [1]. Liver fibrosis is one of the leading causes of morbidity and mortality worldwide. It is caused by various etiologies including autoimmune, viral, metabolic, and cholestatic disease [2]. However, currently little is known about the disorder of immune system involved in hepatic fibrosis.

The liver harbors 80% of all body macrophages [3]. Studies on the liver from rodents showed that about 100 hepatocytes are accompanied by 20 to 40 macrophages [4]. During the past decades, studies have demonstrated that hepatic macrophages perform vital functions in initiating, perpetuating and even limiting inflammation in the liver [5]. Depending on the different types of insults, macrophages acquire a distinct phenotype during a process known as macrophage polarization. During this process, macrophages are either classically activated (M1) or alternatively activated (M2) [6]. However, the molecular mechanism underlying macrophage phenotypic alteration and the effect of these changes on liver fibrosis are not yet clear.

Sympathetic nervous system inhibitors markedly reduce experimentally induced liver fibrosis [7]. β _1_-Adrenergic receptors (β _1_-ARs) were reported to be over-expressed during the process of liver fibrosis [8], suggesting a positive association between β _1_-ARs and liver fibrosis. We and others have found an autoimmune antibody (β _1_-AA), which binds to the second extracellular loop of β _1_-AR (β _1_-AR-EC _II_, 197–223, with 100% homology between human and mouse) [9]. β _1_-AA, which has an agonist-like effect on the β _1_-AR, leads to sustained activation of the receptor [10]. β _1_-AA is associated with various kinds of cardiovascular diseases such as dilated cardiomyopathy [11], atherosclerosis [12], hypertension [13], and arrhythmia [14]. A previous study demonstrated that the positive rates of the β _1_-AA and the antibodies against the hepatitis virus were highly consistent in patients with hepatitis virus and myocarditis [15]. However, it is still unclear whether β _1_-AA affects the liver function. In addition, we have found that β _1_-AA can directly promote the proliferation of RAW264.7 cells, a mouse-derived macrophage cell line, and stimulate TNFα secretion by these cells [16]. However, these effects cannot be caused directly by isoproterenol, a β _1_-AR agonist [17]. Nevertheless, the contribution of macrophages to the hepatic fibrosis induced by β _1_-AA remains unknown.

Herein therefore, we report the effect of macrophage disorder upon liver fibrosis and dysfunctions caused by β _1_-AA, and propose a novel mechanism responsible for the effect of β _1_-AA on hepatic function.

## Materials and Methods

### Antibodies and chemicals

The antibodies used in this study were as follows: anti-CD206 (ab64693; Abcam; Cambridge, UK); anti-CD163 (ab85182; Abcam); anti-MMP2 (4022S; Cell Signaling Technology, Danvers, USA); anti-MMP9 (3852S; Cell Signaling Technology); anti-TNFα (sc-1349; Santa Cruz Biotechnology; Dallas, USA); anti-CD68 (ab53444; Abcam); anti-mouse MHC Class II FITC (558593; BD Biosciences; Franklin Lakes, USA); anti-mouse CD11b Per-Cyanine5.5 (31-1174-00; RevMAb Biosciences, South San Francisco, USA); anti-mouse CD284 (TLR4) Alexa Fluor®488 (53-9041-82; Thermo Fisher Scientific, Waltham, USA); anti-mouse CD14 PerCP-Cy5.5 (45-0141-80; Thermo Fisher Scientific); rat anti-mouse Mannose Receptor Monoclonal Antibody (HM1049; Hycult Biotech, Uden, the Netherlands); anti-cleaved Caspase-3 (9660S; Cell Signaling Technology); anti-β _1_-AR (bs-0498R; Bioss, Beijing, China); and Annexin V-FITC Apoptosis Detection Kit 100 tests Kit Antibody (BMS500FI/100; Thermo Fisher Scientific).

### Study population

Blood samples were collected from 199 patients suffering from primary biliary cirrhosis (PBC), as well as from 71 healthy controls (Ditan Hospital Clinic, Capital Medical University, Beijing, China). PBC was diagnosed when two out of three criteria were satisfied: (1) cholestatic liver biochemistry with an alkaline phosphatase (ALP) level at least 1.5 times higher than the upper limit of the normal range (ULN); (2) anti-mitochondrial antibody (AMA) positivity; (3) histological features “nonsuppurative destructive cholangitis with destruction of interlobular biliary ducts” [18]. Clinical characteristics are summarized in Table 1. The research protocol is in accordance with the Helsinki Declaration of 1975, revised in 1983. It was also approved by the Institutional Committee for the Protection of Human Subjects of the Capital Medical University (No. AEEI-2016-013). All patients were informed of the purpose of the research and both oral and written consents were obtained.

**Table:** **Table 1** Demographic and clinical characteristics of the participants

Variable		Primary biliary cholangitis ( *n*=199)		Controls ( *n*=71)		*P* value
		Available data	Results		Available data	Results		
Age (years)		199	62 (23–81)		71	59 (19–85)		NS
Female sex (%)		199	164 (82)		71	59 (84)		NS
AMA titer		199	1:320 (1:120-1:1000 or higher)		71	Negative		*P*<0.0001
PBC-specific ANAs (%)		32	24(75)		71	Negative		*P*<0.0001
Total bilirubin (μmol/L)		199	39 (4–417)		71	14 (2.3–18.5)		*P*<0.01
ALP (×ULN)		199	151 (74–389)		71	82 (56–134)		*P*<0.01
ALT (×ULN)		199	34 (6–245)		71	24 (7.0–39)		*P*<0.05
Prothrombin index (%)		94	16 (10–31)		45	11.2 (9.6–12)		*P*<0.05
γ-GT		173	97 (10–1274)		54	34 (7.5–44)		*P*<0.01

AMA: anti-mitochondrial antibody; ANA: antinuclear antibody; ALP: alkaline phosphatase activity; ALT: alanine aminotransferas; γ-GT: γ-glutamyltransferase. NS: not significant.

### Animals

The healthy male Balb/c mice used in the study were obtained from the Animal Center of the Capital Medical University (Beijing, China). The animals were housed in pathogen-free facilities at 20°C to 22°C and were exposed to a 12 h light-dark cycle. Animals were fed with animal chow and water *ad libitum* throughout the study. All animal experiments were done in accordance with the Guide for the Care and Use of Laboratory Animals published by the Ministry of the People’s Republic of China (1998) and approved by the Institutional Committee on Animal Care of Capital Medical University.

### *In vivo* immunization with β _1_-AA

To obtain a high-specificity antibody, we designed and synthesized a monoclonal antibody β _1_-AA that could specifically bind to the β _1_-AR-EC _II_ in collaboration with AbMax Biotechnology (Beijing, China). The production of monoclonal β _1_-AA was performed by cross-linked studies using hybridoma cells [19]. The β _1_-AA was used to passively immunize Balb/c mice (5 μg/g body weight) via intraperitoneal injection. A booster immunization was given once every two weeks for 20 weeks. The level of β _1_-AA in mouse blood was detected by enzyme-linked immunosorbent assay (ELISA) as described previously [ 20, 21] . A group of serum proteins, including alanine aminotransferase (ALT), aspartate transaminase (AST), albumin (ALB) and globulin (GLB), were used as immunization indicators and quantified with an automatic biochemical analyzer (Abbott, Abbott Park, USA). The left ventricular ejection fraction (LVEF) and left ventricular end-diastolic dimension (LVEDD), which are indicators of heart function, were monitored by echo cardiography (Vevo2100; Visual Sonics, Toronto, Canada).

### Cell culture

Murine macrophage-like RAW264.7 cell line and liver QSG-7701 cell line were obtained from the Cell Resource Center of the Chinese Academy of Medical Sciences (Beijing, China). The cells were maintained in Dulbecco modified Eagle’s medium (DMEM; Thermo Fisher Scientific) containing 10% FBS (Excell Bio, Suzhou, China) and 1% Penicillin-Streptomycin Solution (Solarbio, Beijing, China) at 37°C with 5% CO _2_.

### Enzyme-linked immunosorbent assay (ELISA)

The titer of β _1_-AA was measured by ELISA [16], and the results were expressed as absorbance values. The absorbance values were measured at 405 nm (A405) using a microplate reader (Spectra Max Plus; Molecular Devices, Sunnyvale, USA). We also calculated the positive/negative (P/N) ratio of each sample: P/N= (A405 _specimen_– A405 _blank_) / (A405 _negative control_– A405 _blank control_)]. Those samples with a P/N ratio ≥ 2.1 were considered β _1_-AA positive.

### Western blot analysis

Total protein was isolated from cells or tissues with lysis buffer (APPLYGEN, Beijing, China). Protein was prepared as described previously. And 30–70 μg of protein per sample was separated via gel electrophoresis, transferred to a poly-vinylidene fluoride membrane, and blocked with 5% milk. The membrane was probed with primary antibodies overnight at 4°C, then incubated with secondary HRP-conjugated antibody at RT and exposed to enhanced chemiluminescent substrate.

### Real-time PCR

Total RNA was extracted from the frozen liver tissues with Trizol reagent (Thermo Fisher Scientific). The RNA concentration and purity were determined by measuring A260/A280. The target genes such as: *Collagen I*, *Collagen III*, *MMP2* and *MMP9* were amplified using SYBR® Premix Ex (Thermo Fisher Scientific) according to the manufacturer’s instructions. The amplification was performed under the following thermal conditions: initial denaturation at 95°C for 10 s, 40 cycles of denaturation at 95°C for 5 s, annealing at 60°C for 20 s. The dissociation curve was performed at 95°C for 1 min, 55°C for 30 s, and 95°C for 30 s. The expressions of target genes in the experimental group were compared to those in the control group (relative gene expression). The qRT-PCR gene expression results were normalized using the reference gene *GAPDH* and calculated using the 2 ^−ΔΔCt^ method. The primer sequences used in this study are shown in Table 2.

**Table:** **Table 2** Sequences of primers used for real-time PCR

Gene	Primer sequence (5′→3′)
Col1a2-F	AGTCGATGGCTGCTCCAAAA
Col1a2-R	AGCACCACCAATGTCCAGAG
Col3a1-F	AGGAGAACCACTGTTGCCTG
Col3a1-R	AGGAGAACCACTGTTGCCTG
MMP2-F	ACCCAGATGTGGCCAACTAC
MMP2-R	AAAGCATCATCCACGGTTTC
MMP9-F	TGTACCGCTATGGTTACACTGC
MMP9-R	GGCAGGGACAGTTGCTTCT
GAPDH-F	GGTTGTCTCCTGCGACTTCA
GAPDH-R	GGTGGTCCAGGGTTTCTTACTC

### Migration assay

For the scratch wound assay, 2×10 ^5^ cells/well (three replicates per group) were plated into a 12-well plate and incubated to reach 80% confluence. The monolayer was scratched using a tip and washed with serum-free medium to remove detached cells. Then the cells were cultured in complete medium supplemented with different β _1_-AA concentrations (10 ^–6^ M, 10 ^–7^ M, and 10 ^–8^ M) or fresh medium alone. RAW264.7 cells were photographed at 0 h and 24 h post-wounding with an invert microscope (Axio Vert A1; ZEISS, Oberkochen, Germany).

For the transwell assay, 1×10 ^4^ cells/well (three replicates per group) were suspended in low serum (5% FBS) medium and seeded into the upper chamber of transwell 24-well plates (Corning, Corning, USA) with 8 μm pore filters. Then the lower chamber was added with complete medium (containing 10% FBS) supplemented with different β _1_-AA concentrations (10 ^–6^  M, 10 ^–7^  M, and 10 ^–8^  M). After 12 h, the cells attached on the upper surface of the filter membranes were cleaned and migrated cells of the lower surface were stained with 0.5% crystal violet for several minutes. The level of migration was observed under a light microscope with ZEN Digital Imaging (ZEISS).

### Appoptosis detected by TUNEL assay and Annexin V/PI staining

An *in situ* apoptosis detection kit (Roche, Basel, Switzerland) was used to assess the apoptosis level of hepatic tissue. Briefly, the liver tissue blocks were fixed with a 4% paraformaldehyde PBS solution and then embedded in paraffin. TUNEL staining was performed on the paraffin slides as described in the manufacturer’s protocol. The apoptosis index was estimated as a percentage of apoptotic nuclei to total nuclei on each slide.

Cell apoptosis was detected by Annexin V/PI staining kit (BD Bioscience, San Jose, USA). Liver QSG-7701 cells were resuspended in 1× binding buffer at a concentration of about 1×10 ^6^ cells/ml, preparing a sufficient volume to have 100 μL per sample. And 5 μL of Annexin V and 10 μL of PI were added to each sample and gently swirled to mix. Then the mixture was incubated for 20 min at room temperature in the dark. Then 400 μL 1× binding buffer was added to each sample, and gently mixed or flicked. And the cells were immediately (within 1 h) analyzed by flow cytometry (Guava Easy incite, Millipore, USA).

### Flow cytometry

RAW264.7 cells were plated at 2×10 ^5^ cells/well into a 6-well plate and incubated overnight. Then the cells were cultured in complete medium supplemented with different β _1_-AA concentrations (10 ^–6^ M, 10 ^–7^ M, and 10 ^–8^ M) or fresh medium alone. Cells were collected and washed 3 times with PBS. Cells were resuspended and incubated with FITC-MHCII, PE-CD14, PE-CD11b, PECy5.5-CD206 and FITC-TLR4 for 30 min at 4°C in the dark. Cells were centrifuged and washed 3 times with PBS. Finally, 500 μL of PBS was added to each tube to resuspend the cells, and the fluorescence intensity of cells was measured by the flow cytometry.

### Protein-antibody array

The protein-antibody array was used to quantitatively compare inflammatory cytokines in the peripheral serum obtained from the β _1_-AA-induced hepatic fibrosis model compared to that of controls. The Mouse Inflammation Array 1 kit (QAH-INF-1; RayBiotech, Norcross, USA) was used for the simultaneous analysis of 308 selected cytokines. According to the manufacturer’s instructions, the tested samples were dialyzed in PBS first and then biotinylated. Spin column was used to dialyze biotinylated samples. Then samples were incubated with streptavidin-conjugated fluor. The signals were captured using a fluorescent dye conjugated with streptavidin (cy3 equivalent) and were visualized with a GenePix 4000B system (Axon Instruments, Foster City, USA). GenePix Pro 6.0 software (Axon Instruments) was used for densitometry analysis ( www.raybiotech.com).

### Phagocytosis assay

RAW264.7 cells were plated on a 4-well chamber slide and allowed to adhere overnight. The latex beads-Rabbit IgG-FITC complex (Cayman Chemical, Ann Arbor, USA) was added directly to the culture medium (1:200) and incubated at 37°C for 2 h. After two washes with the assay buffer, the cells were visualized at a magnification of 20×using the light microscope with ZEN Digital Imaging.

### TNF-α and ROS level detection

TNF-α in the supernatant of the RAW264.7 cells treated by β _1_-AA was quantified using the TNF-α ELISA kit (Boster, Pleasanton, USA) according to the manufacturer’s protocol. The intracellular ROS level in macrophages was quantified by using the 2,7-dichlorofluorescein diacetate (DCFH-DA; Sigma, St Louis, USA). The macrophages were seeded in 6-well plates at 1.6×10 ^5^ cells/well and allowed to adhere overnight. Subsequently, the medium was replaced by fresh medium with different β _1_-AA concentrations (10 ^–6^ M, 10 ^–7^ M, and 10 ^–8^ M), negative IgG (10 ^–7^ M), metoprolol (3× 10 ^–7^ M), LPS (10 ng/mL; Sigma-Aldrich) or fresh medium alone and incubated for 24 h. The plates were washed twice with PBS and incubated with DCFH-DA (10 μM) at 37°C for 20 min. The mean fluorescence intensity (MFI) of the cells was determined at the best excitation wavelength of 485 nm and emission wavelength of 525 nm using the microplate reader.

### Histological and confocal microscopic analysis

For histologic analysis, livers were fixed with 4% paraformaldehyde. Fixed livers were embedded in paraffin and cut transversely into 5-μm sections. Serial liver sections were fixed with 4% paraformaldehyde for 15 min, permeabilized with 0.1% Triton X-100 in PBS for 10 min, blocked with 5% BSA for 1 h, and incubated with anti-HNF4α antibody (1:200 dilution) overnight at 4°C, and subsequently with tetramethyl rhodamine (TRITC)-conjugated anti-rabbit IgG (1:150 dilution). After wash with PBS, coverslips were mounted using an anti-fade solution (KPL, Gaithersburg, USA). The negative control (without primary antibody) was stained and processed in parallel. Fluorescent images were acquired by using the Fluoview software (Olympus, Tokyo, Japan) via the FV3000 confocal microscope (Olympus).

### Statistical analysis

As the clinical samples data had normal distribution, thus a Student’s *t*-test was used to analyze differences between the PBC patients and the healthy controls. In the mouse study, groups of 6 to 8 mice were compared using One-way ANOVA. *In vitro*, each experiment was performed 4 to 5 times. All results were presented as the mean±SEM and were subsequently assessed by the Bonferroni test using Prism 6.0 software (GraphPad, San Diego, USA) except for the ones of the protein array assay. *P*<0.05 was considered statistically significant. The results from the protein array were analyzed in fold change of β _1_-AA vs Vehicle. Fold changes of>2.0 or<0.5 were considered statistically significant.

## Results

### Long-term existence of β _1_-AA induces hepatic fibrosis

**Figure 1:**
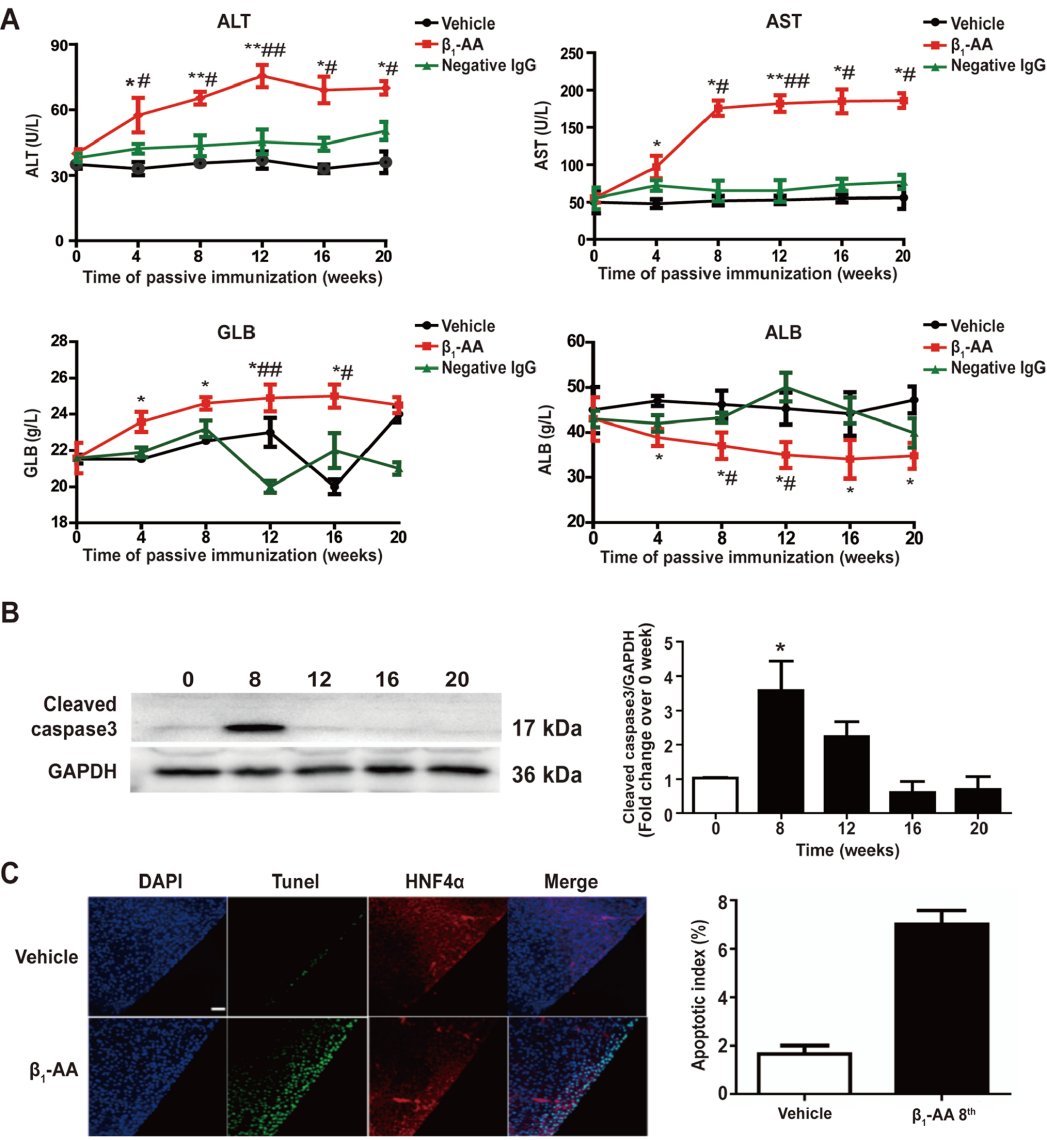
Figure 1 Long-term existence of β1-AA induces hepatic fibrosis (A) Changes in the serum levels of ALT, AST, ALB and GLB at different periods of passive immunization. (B) The expression level of cleaved Caspase 3 at different time points. (C) The detection of the co-localization of HNF4α (the hepatocyte-specific marker) with apoptosis markers in liver tissue by TUNEL staining and confocal microscopy. Scale bar: 50 μm. n=8. * P<0.05, ** P<0.01 vs Vehicle group (A,C) or 0 week (B); # P<0.05, ## P<0.01 vs Negative IgG group.

To investigate the effect of β _1_-AA on the liver, a passive immunization mouse model was established. Our results demonstrated that the level of β _1_-AA remained high in mice over time, suggesting a passive immunization mouse model was successfully established ( Supplementary Figure S1). A group of serum proteins were used as immunization indicators and quantified. These protein indicators included alanine aminotransferase (ALT), aspartate transaminase (AST), albumin (ALB), and globulin (GLB). As shown in Figure 1, a significant increase in the ALT, AST and GLB levels were observed at the 4 ^th^ week after passive immunization with β _1_-AA. Meanwhile, the serum albumin level was decreased throughout the entire period of immunization ( Figure 1A). However, the creatine kinase-MB (CK-MB) and lactate dehydrogenase (LDH) were not increased ( Supplementary Figure S2A). Furthermore, the left ventricular ejection fraction (LVEF) and left ventricular end-diastolic dimension (LVEDD), which are indicators of heart function, showed no significant differences between groups at this time point ( Supplementary Figure S2B). These results suggested that β _1_-AA-induced liver injury might occur earlier and independently of heart dysfunction.

To assess hepatic injury during β _1_-AA-induced hepatic fibrosis, western blot analysis and hepatic apoptosis assay were performed. Western blot analysis results showed that cleaved caspase3 protein was greatly increased at the 8 ^th^ week in the β _1_-AA immunization group ( Figure 1B). Simultaneously, TUNEL staining showed that the number of positive cells in the β _1_-AA immunization group was higher than that of the vehicle group, and confocal microscopy analysis also showed that HNF4α co-localized with TUNEL-positive cells ( Figure 1C). These results suggested that the hepatocytes suffered apoptosis due to continued exposure to β _1_-AA.

**Figure 2:**
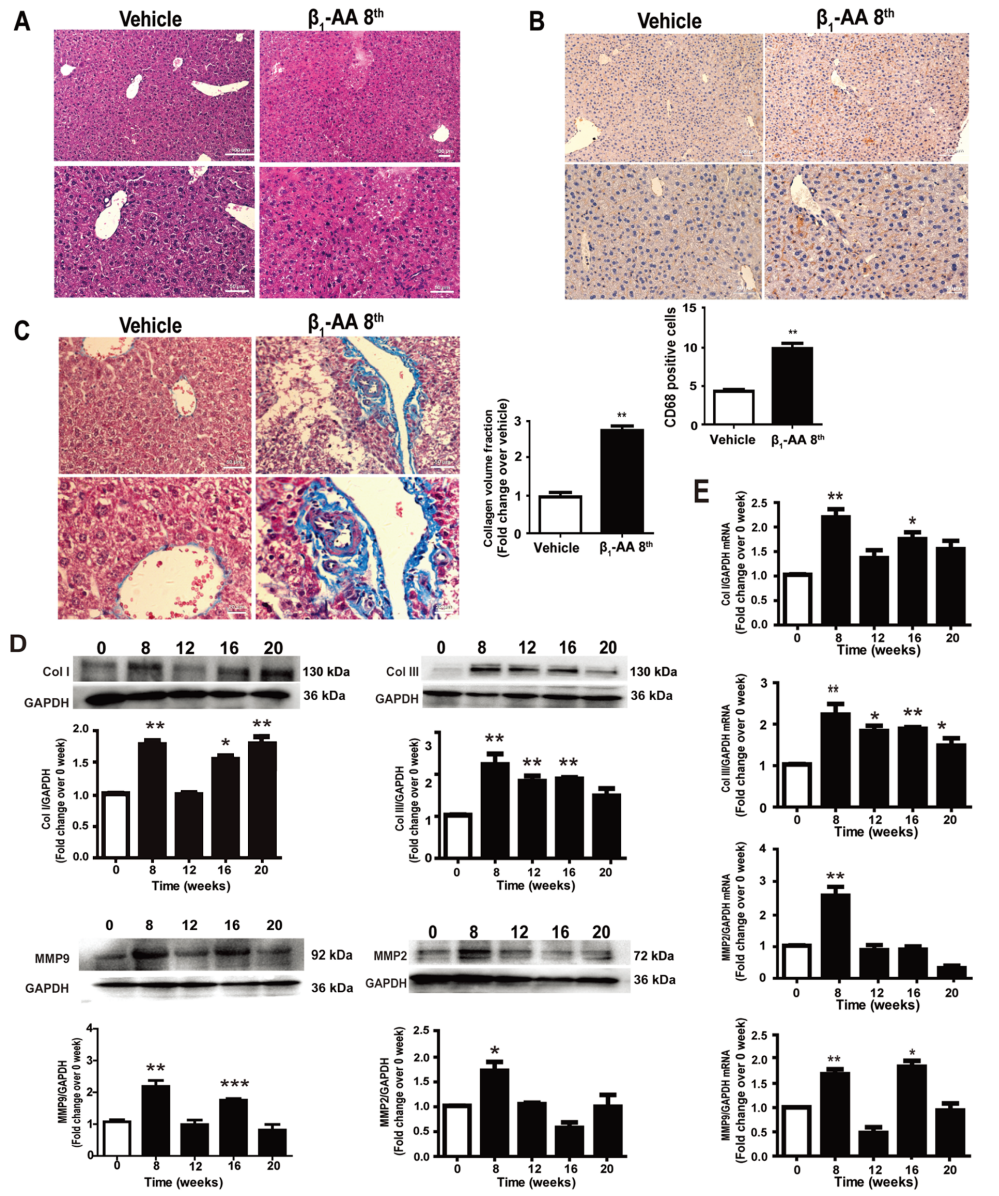
Figure 2 Hepatic injury and collagen deposition are induced by β _1_-AA (A) Hematoxylin-eosin liver staining showed no damage in the nuclei and cytoplasm in the hepatocytes of the vehicle group, whereas cell damage and centrilobular congestion in the β 1-AA immunization group were observed at the 8 th week after the beginning of the exposure. Scale bar: 100 μm (top); 50 μm (bottom). (B) Detection of the expression of CD68-positive macrophages in mouse liver tissue. Scale bar: 100μm (top); 50μm (bottom). (C) Masson staining was used to detect collagen deposition around blood vessels in liver tissue. Scale bar: 50 μm (top); 20 μm (bottom). (D) Detection of protein levels of liver fibrosis-related markers in mice. (E) Detection of mRNA levels of liver fibrosis-related marker genes in mice. n=8. * P<0.05, ** P<0.01 vs Vehicle (B,C) or 0 week (D,E).

Hematoxylin-eosin liver staining showed no damage in nuclei and cytoplasm in the hepatic cells of the vehicle group, whereas, cell damage and centrilobular congestion in the β _1_-AA immunization group were observed at the 8 ^th^ week post-exposure ( Figure 2A). Studies have reported that Kupffer cells play a role in hepatic injury and fibrosis [ 22, 23] . Our immunohistochemistry results suggested that the hepatocytes of β _1_-AA group contained a significantly higher number of CD68-positive macrophages than those of the vehicle control mice at the 8 ^th^ week ( Figure 2B). Masson staining analysis demonstrated that liver collagen deposition was also significantly larger in the β _1_-AA group at this time point ( Figure 2C). Meanwhile, there was a significant increase in protein ( Figure 2D) and mRNA ( Figure 2E) levels of the markers associated with fibrosis such as Collagen I, Collagen III, MMP2 and MMP9 at the 8 ^th^ week after β _1_-AA passive immunization. These results suggested that liver injury and fibrosis might be induced by long-term exposure to β _1_-AA.

### Long-term exposure to β _1_-AA induces dysfunctionin macrophages

**Figure 3:**
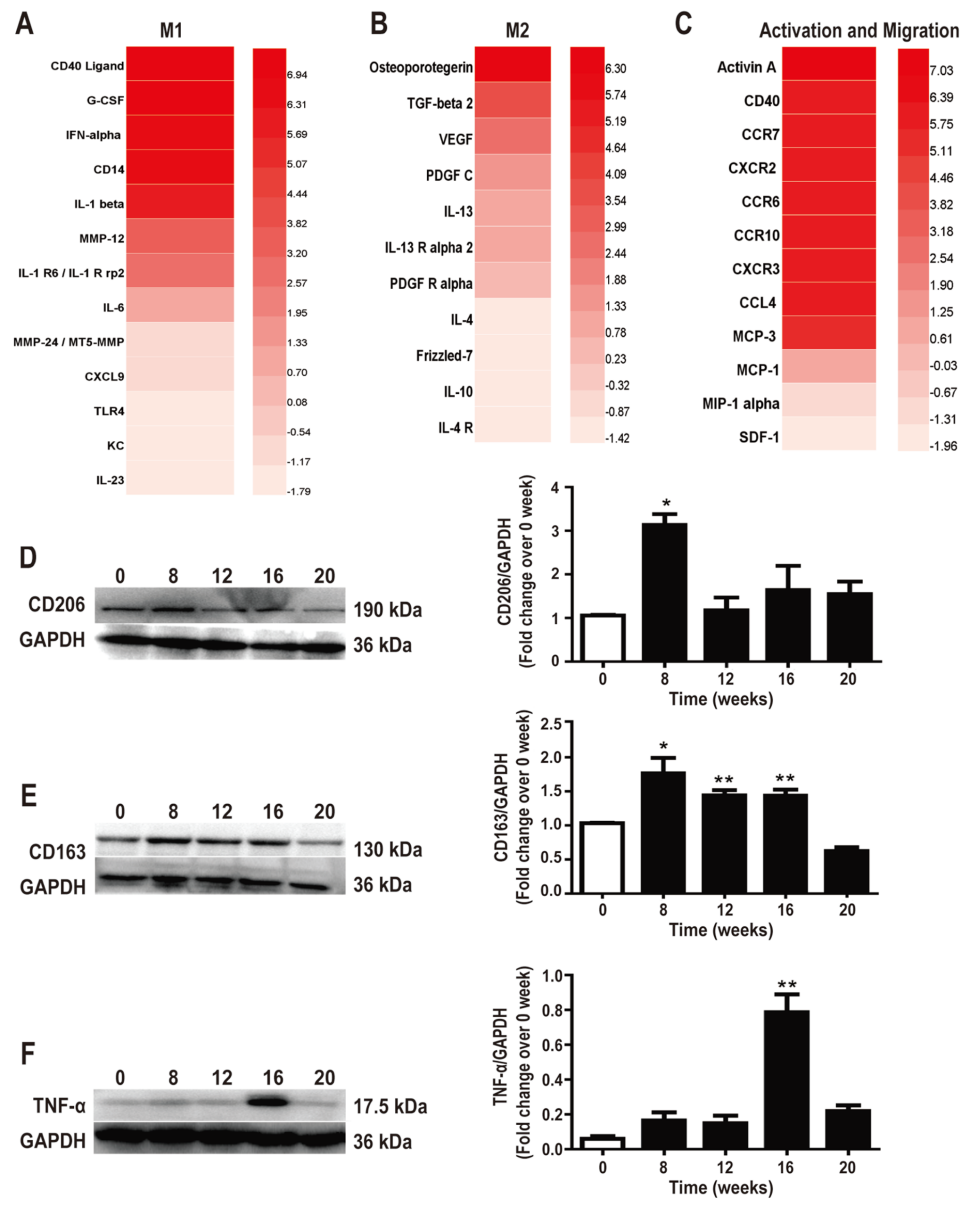
Figure 3 Long-term exposure to β _1_-AA induces disordered functions in macrophages (A-C) Protein-antibody array results show the changes of cytokines related to phenotypic transformation, activation and migration of macrophages under long-term exposure to β 1-AA. (D) The expression of CD206 after immunization. (E) The expression of CD163 after immunization. (F) The expression of TNF-α after immunization. n=8. * P<0.05, ** P<0.01 vs 0 week.

In order to assess cytokine-related secretory changes in macrophages stimulated by β _1_-AA *in vivo*, we identified the presence of 308 cytokines in the sera of β _1_-AA-immunized mice using a protein array. A significant change was found in cytokines associated with the macrophage phenotype, migration, and activation ( Figure 3A–C). In addition, western blot analysis showed that the M2 macrophage markers, CD206 and CD163, were upregulated at the 8 ^th^ week ( Figure 3D,E) **,** whereas the M1 macrophage marker, TNFα, was upregulated at the 16 ^th^ week of the β _1_-AA-induced hepatic fibrosis ( Figure 3F). These results suggested that long-term exposure to β _1_-AA changed the phenotype of hepatic macrophages.

### β _1_-AA induces RAW264.7 cells to differentiate intoM1 macrophages

**Figure 4:**
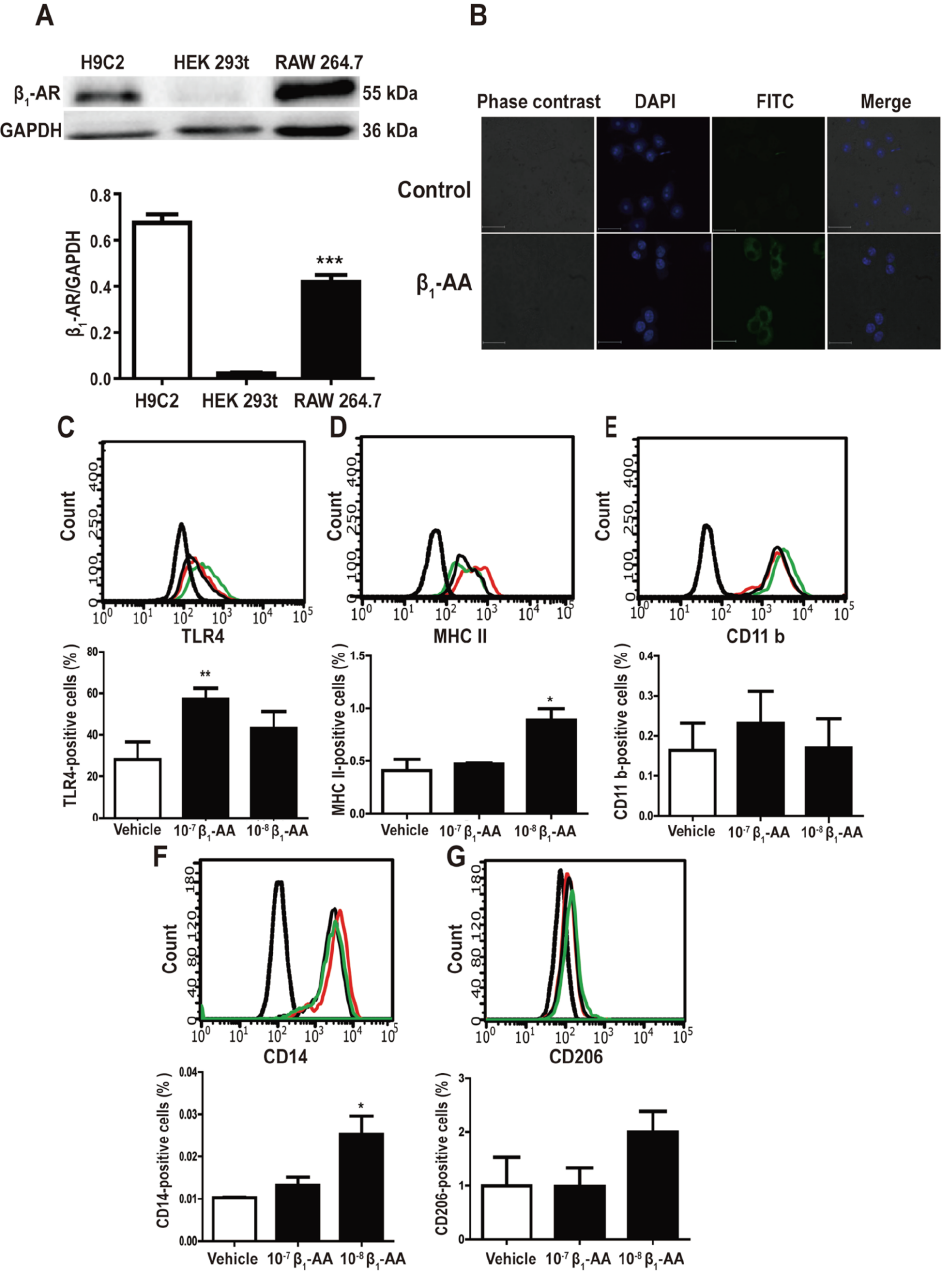
Figure 4 β _1_-AA induces RAW264.7 cells to differentiate into M1 macrophages (A) The expression of β 1-AR in RAW264.7 cells. *** P<0.001 RAW264.7 vs HEK293. (B) Immunofluorescence shows that β 1-AA can bind with β 1-AR on the surface of RAW264.7 cells. (C-G)M1 macrophage markers(TLR4, MHCII, CD11b, CD14, CD206)on the cell surface analyzed by flow cytometry. Data are presented as the mean±SD of three independent experiments. * P<0.05, ** P<0.01, β 1-AA group vs Vehicle group.

To assess the effect of β _1_-AA on macrophages directly, we used the RAW264.7 cells for *in vitro* study. Western blot analysis and immunofluorescence assay results showed that β _1_-AR was expressed on the RAW264.7 cells and could bind with β _1_-AA ( Figure 4A,B).

M1 and M2 phenotypes represent the two phenotypic extremes of the macrophage activation spectrum under different environmental signals. M1 macrophages promote inflammatory reactions, whereas M2 macrophages regulate immunological responses and enhance wound healing [24]. Here, we questioned whether β _1_-AA directs macrophages to acquire a specific activation phenotype. The poly-specific receptor (CD14), the member of the toll-like receptor family (TLR4), the integrin family member (CD11b), the mannose receptor (MR, also known as CD206), and the main histo-compatible complex II (MHCII) were selected as M1 macrophage type and quantified by flow cytometry. The results indicated that treatment with β _1_-AA (10 ^–7^ M) upregulated the protein level of TLR4. In addition, protein levels of MHCII and CD14 were increased by incubation with β _1_-AA (10 ^–8^ M). On the contrary, CD206, one of the M2 macrophage markers, showed no altered expression after β _1_-AA (10 ^–8^ M) treatment ( Figure 4C–G). Collectively, these results suggested that β _1_-AA activated macrophages in the classical way, which implies that macrophage cells perceive β _1_-AA as a dangerous antigen.

### β _1_-AA induces functional changes in RAW264.7 cells

**Figure 5:**
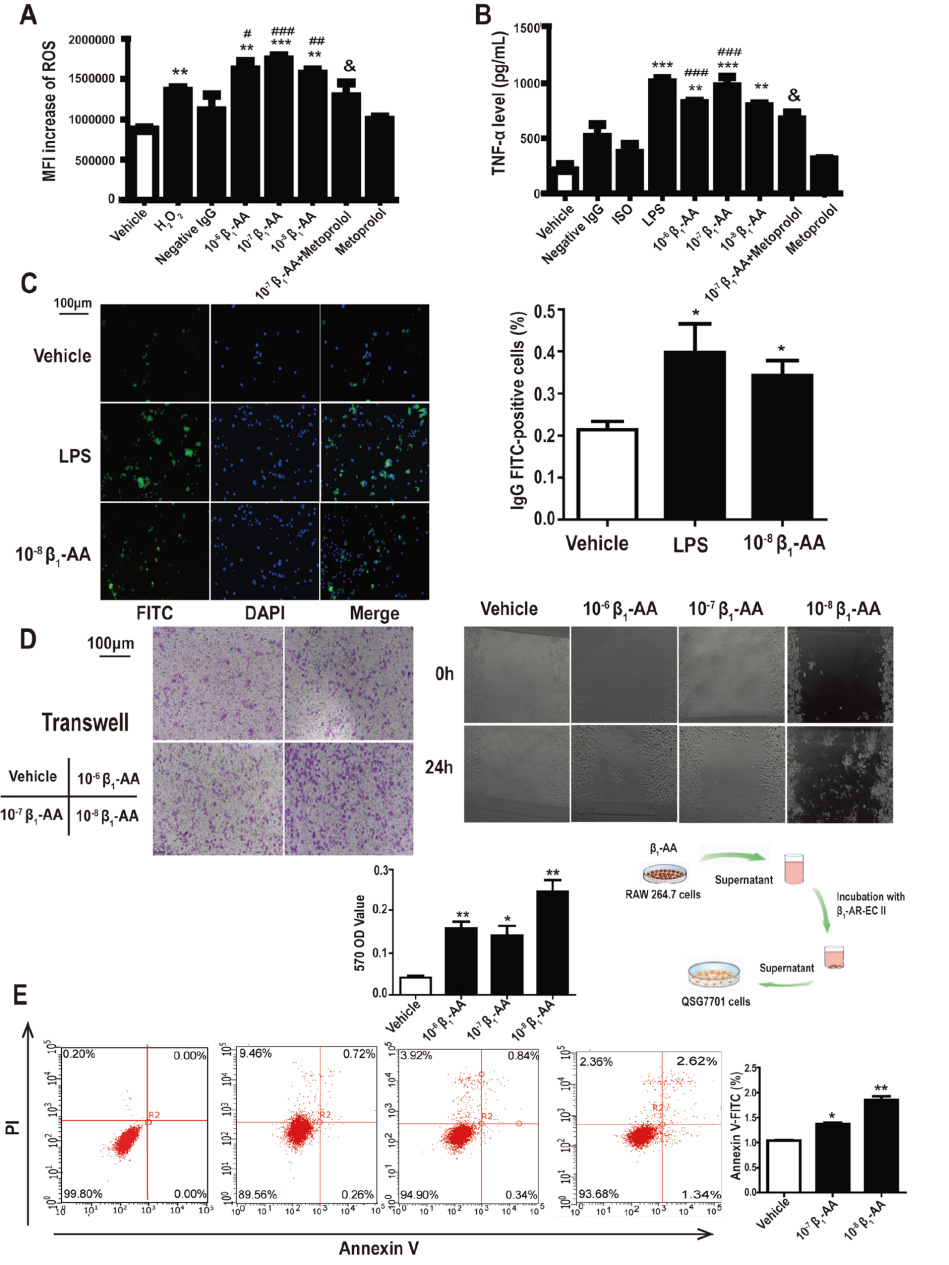
Figure 5 β _1_-AA induces functional changes of RAW264.7 cells Different concentrations of β 1-AA stimulate RAW264.7 cells to produce ROS. (B) Different concentrations of β 1-AA promoted TNF-α secretion. (C) The phagocytosis of macrophages stimulated by β 1-AA or LPS analyzed by immunofluorescence microscopy. Scale bar: 100 μm. (D) The effect of β 1-AA stimulation on the migration of macrophages. Scale bar: 100 μm. (E) Supernatant from β 1-AA-stimulated macrophages induces apoptotic cell death in hepatocytes, as determined by flow cytometry with Annexin V/ PI staining. Representative scatter plots of PI ( y axis) vs Annexin V ( x axis) are shown. The lower right quadrants represent Annexin V-positive and PI-negative apoptotic cells. Data are presented as the mean±SD of three independent experiments. * P<0.05, ** P<0.01, *** P<0.001 vs Vehicle group; * P<0.05, ** P<0.01, *** P<0.001 vs Negative IgG group; & P<0.05 vs 10 –7 β 1-AA group.

Phagocytosis by macrophages leads to the “respiratory burst” response that in turn increases the production of ROS [25]. Therefore, the level of ROS in macrophages was used as an index of RAW264.7 cell activation. The results showed that LPS (10 ng/mL) greatly stimulated ROS generation in macrophages. Furthermore, the exposure of macrophages to various concentrations of β _1_-AA (10 ^–6^ M, 10 ^–7^ M and 10 ^–8^ M) also resulted in increased ROS production, compared to negative IgG control. The effect of β _1_-AA on ROS was partially reversed by the addition of the β _1_-AR blocker, metoprolol (3×10 ^–7^  M). Similarly, our results demonstrated that LPS (10 ng/mL) stimulated TNF-α secretion in RAW264.7 cells, and exposure to various concentrations of β _1_-AA (10 ^–6^ M, 10 ^–7^ M and 10 ^–8^ M) also promoted TNF-α secretion. However, the β _1_-AR agonist, isoproterenol, had no obvious effect on TNF-α secretion in RAW264.7 cells ( Figure 5A,B).

The results from the phagocytosis assay showed that 24 h of treatment with β _1_-AA (10 ^–8^ M) caused the same increase in phagocytosis as 24 h of treatment with LPS (10 ng/mL). Wound healing and transwell assays showed that different concentrations of β _1_-AA (10 ^–6^ M, 10 ^–7^ M and 10 ^–8^ M) induced RAW264.7 cell migration **(** Figure 5C,D **)**. These results suggested that β _1_-AA exposure increases macrophage dysfunction.

QSG7701 cells were incubated with the supernatant from β _1_-AA-treated RAW264.7 cells, and the apoptosis level of QSG7701 cells was measured by Annexin V/PI. As shown in Figure 5E, the apoptosis rate of QSG7701 cells was increased when hepatocytes were stimulated for 24 h. The results implied that β _1_-AA-activated macrophages might lead to hepatocyte injury.

### Serum β _1_-AA level is markedly increased in primary biliary cholangitis patients

**Figure 6:**
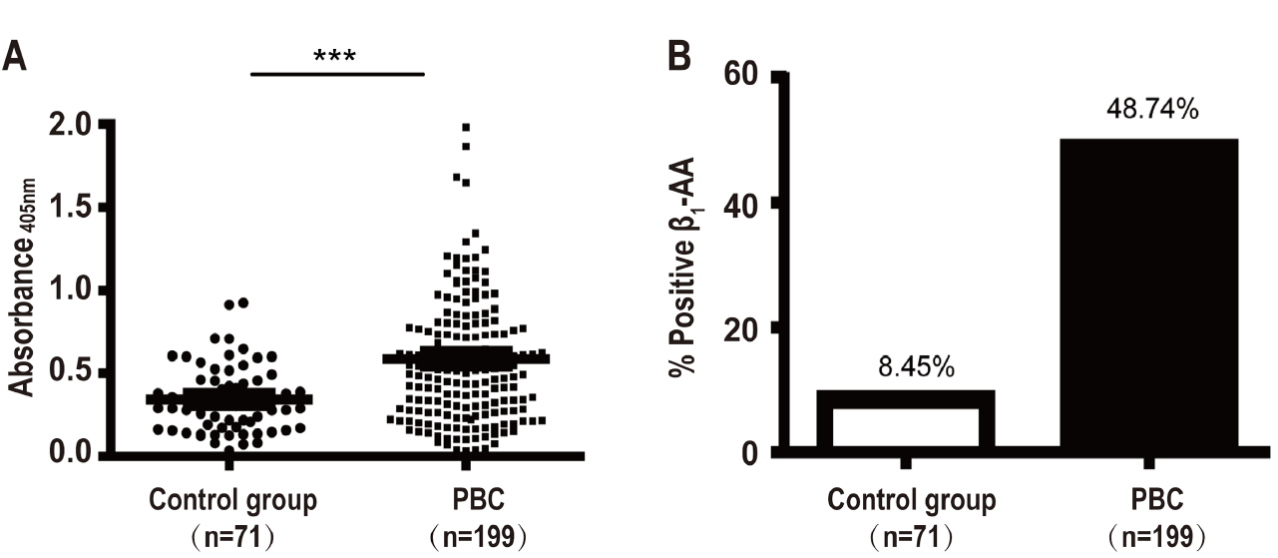
Figure 6 Serum β _1_-AA level is markedly increased in PBC compared to that in controls (A) Compared with that in the normal controls ( n=71), the OD value of β 1-AA in the serum of patients with PBC was significantly increased. (B) The positive rate of β 1-AA is higher in the serum of patients with PBC ( n=199) than in the serum of normal controls. *** P<0.001.

An increased serum level of β _1_-AA was found in the primary biliary cholangitis (PBC) patients compared to that in the controls, represented by an absorbance value of 0.589±0.436 in the PBC group which was higher than that (0.343±0.199) in the control group ( Figure 6A **).** Furthermore, from the 199 samples of patients in the PBC group, 97 patients were β _1_-AA-positive, indicated by absorbance of sample/absorbance reference ratio ≥ 2.1, and 102 were β _1_-AA-negative. Whereas, within the control group, 6 patients were β _1_-AA-positive and 65 were β _1_-AA-negative. The positivity rate of β _1_-AA in PBC patients (48.74%) was significantly higher than that (8.45%) of the controls **(** Figure 6B). Taken together, these data suggest that β _1_-AA is related to PBC.

## Discussion

In the present study, we attempted to link, for the first time, the incidence of PBC to the level of β _1_-AA. We used human and mouse samples, as well as *in vitro* cell culture experiments to demonstrate that liver fibrosis and injury are induced by long-term exposure to β _1_-AA. Meanwhile, the number of M2 macrophages is significantly increased in hepatic fibrosis, suggesting that a phenotype change in macrophages occurs during β _1_-AA-induced fibrosis.

Previous studies have demonstrated that β _1_-AA plays a role in the development of cardiovascular diseases. When we constructed the β _1_-AA passive immunization model and observed its long-term effects on mouse cardiac function, we unexpectedly found that the long-term existence of high levels of β _1_-AA may cause liver damage, and this damage occurs before the damage of heart functions. However, it is not clear whether β _1_-AA in the circulating blood of clinical patients is related to liver dysfunction. In this research, we selected the PBC disease to assess the contribution of β _1_-AA to liver diseases. PBC, which is one of the most common autoimmune liver diseases [26], is characterized by the existence of AMAs and destruction of intrahepatic small bile ducts, leading to cholangitis, fibrosis and potentially cirrhosis [27]. The liver is an important organ involved in the immune function. Nevertheless, β _1_-AA is produced by the immune system, and its relationship with liver disease causes great concern. Our study showed that the PBC group had significantly higher level of β _1_-AA than the control group, thus necessitating the investigation of the levels of β _1_-AA in other chronic liver diseases in future studies. However, this study cannot explain the source of β _1_-AA in the diseases ( *e.g.*, PBC). At present, we are planning to establish a bile duct ligation mouse model to further explore the source of β _1_-AA in the PBC model.

In this study, we first found that the long-term existence of β _1_-AA caused liver dysfunction and induced hepatocyte apoptosis in mice. On this basis, HE staining and immunohistochemical staining showed that a large number of hepatocyte damage, central lobular congestion and macrophage infiltration occur in the liver tissues of β _1_-AA-passively-immunized mice. Combined with Masson staining, western blot analysis and PCR experiments, we confirmed that there is a large amount of collagen deposition in liver tissue, suggesting that long-term exposure to β _1_-AA may cause liver damage and fibrosis. Previous studies have demonstrated that multiple cell populations including hepatic macrophages, B cells, T cells and HSCs influence the development of liver fibrosis. Among these cells, macrophages exert a profound effect on HSCs and hepatic fibrosis [23]. However, the specific mechanism underlying macrophage function is not very clear yet. Herein, we verified that macrophage phenotype changes induced by β _1_-AA lead to liver fibrosis. By immunohistochemistry analysis, we showed that the level of CD68, which is a marker for Kupffer cells, was higher in the passive immunization group than the vehicle control group at the 8 ^th^ week. Meanwhile, western blot analysis results showed that CD206 and CD163, both of which are associated with M2 macrophages, were significantly increased in the β _1_-AA-induced hepatic fibrosis model, overlapping with the phase of hepatic fibrosis. These results are consistent with those obtained with mice hepatic fibrosis model reported by others [28]. It was reported that the number of pro-inflammatory M1 macrophages was increased first during the acute inflammatory response [29]. However, in our β _1_-AA-induced hepatic fibrosis model, there was an increase in the expression of M1 marker at the 16 ^th^ week which is beyond the normal time window for the acute inflammatory response. In order to explain these discrepancies, we proposed that with the exposure to β _1_-AA the liver might set the first acute inflammatory response within a month. Thus, the time point of the 8 ^th^ week after immunization might be too late, which missed the significant inflammatory changes that might have occurred within the first one month of immunization. Despite the liver’s strong compensatory function, it could not effectively recover its function damage caused by long-term exposure to β _1_-AA. Under this scenario, the liver could re-initiate an inflammatory response, causing aggravated liver injury and fibrosis. This hypothesis, however, needs further investigation.

In order to assess β _1_-AA-induced changes in the phenotype of macrophages, we used mouse-derived macrophage cell line, RAW264.7 cells, for *in vitro* studies. *In vitro* experiments showed that β _1_-AA directly caused changes in the polarization, activation, migration and phagocytic function of macrophages. In addition, β _1_-AA stimulated macrophages to secrete a large amount of ROS and TNF-α, and supernatant from β _1_-AA-stimulated macrophages induced hepatocyte apoptosis. We suggest that β _1_-AA may induce hepatocyte damage by inducing the polarization and functional changes of macrophages, and ultimately leads to liver fibrosis and dysfunction. Meanwhile, our results showed that the markers for M1 macrophages, such as TLR4, MHCII and CD14 (Affymetrix analysis), were significantly increased after 24 h of β _1_-AA stimulation, whereas CD11b was increased only marginally. However, the expression level of the marker for M2 macrophages, CD206, did not change. CD14 is located mainly on the surface of monocytes and macrophages, presenting an invading substance to the TLR complex and activating other signaling pathways inside the cell [30]. Toll-like receptors (TLRs) recognize pathogen-derived macromolecules and play an important role in macrophage activation [31]. MHCII predominantly plays a role in the process of phagocytosis, in which foreign substances or antigens are cleaved into peptides in the cell and delivered to the surface of the cell [32]. As a pattern recognition receptor, CD11b is involved in recognizing and binding to specific molecules found on the surface of bacteria or foreign cells. In our study, we found that the expression of CD206 was increased at the 8 ^th^ week in the β _1_-AA passive immunization model. The *in vivo* results are not consistent with the results obtained in the *in vitro* experiments. Possible explanations for these discrepancies might be: (1) in the *in vivo* experiment, unlike in the *in vitro* experiment, the interaction between macrophages and other relevant cells in the liver cannot be ruled out; (2) the amount of β _1_-AA acting on the liver *in vivo* after passive immunization might not have been cleared, whereas the β _1_-AA added to RAW264.7 cells *in vitro* might have been cleared quickly. Therefore, we propose that different β _1_-AA doses may also lead to the inconsistency of the results between the *in vitro* and the *in vivo* studies.

To further determine the mechanisms responsible for β _1_-AA- induced liver fibrosis, we took the serum of immunized mice at 8 ^th^ week for cytokine array detection and compared the levels of more than 300 cytokines secreted by BALB/c mice in the presence or absence of β _1_-AA. We unexpectedly found that the changes in hepatocytes were not the most obvious, but the types of cytokines associated with immune cells changed significantly. In our previous studies, we found that the ratio of CD4 ^+^/CD8 ^+^ T cells is increased during heart failure in rats and β _1_-AA has a profound impact on macrophages, which can directly activate resting macrophages and induce macrophages to secrete large amounts of cytokines [ 33, 16] . Combining with the above results, we believe that β _1_-AA-induced macrophage disorder may play an important role in β _1_-AA-mediated liver fibrosis, so we first focused on macrophages. In this study, only peripheral blood was analyzed by cytokine array, the macrophages located in tissues were not investigated. Hence, we observed tissue macrophage phenotypic change using the β _1_-AA-induced hepatic fibrosis mouse model. We found that liver macrophages phenotypically changed during β _1_-AA-induced fibrosis. Since our results strongly suggested that β _1_-AA could lead to the occurrence of hepatic fibrosis, we investigated changes in the expressions of fibrosis-related marker genes by microarray. The results showed that the expressions of fiber-related Snail1 [34], Acta2 [35], TGFβ1 and many other genes ( Supplementary Figure S3) were increased significantly after β _1_-AA exposure. Furthermore, it has been suggested that macrophage engulfment of apoptotic bodies promotes inflammation and fibrogenesis [36], and we found that macrophages’ phagocytosis was increased by β _1_-AA, implying that the phagocytosis of macrophages induced by β _1_-AA may contribute to hepatic fibrosis. In the future, we plan to further investigate the mechanism of macrophage phenotype conversion and the role of macrophages in β _1_-AA-induced hepatic fibrosis by liver macrophage depletion.

It is well known that β _1_-AA exerts its biological effects by binding to β _1_-AR on the cell surface. Therefore, in order to determine whether β _1_-AA could directly stimulate hepatocytes and induce hepatocyte apoptosis and HSC activation, we first detected the expression of β _1_-AR in liver tissues. Although we found that there is only a small amount of β _1_-AR on the surface of liver cells ( Supplementary Figure S4), it is theoretically possible that β _1_-AA may bind to β _1_-AR and cause hepatocyte damage. Therefore, we have designed a new research plan to observe the direct effects of β _1_-AA on hepatocytes and HSCs.

In conclusion, our study is the first to report the direct effect of β _1_-AA on the liver disease, and this effect is early and independent of heart dysfunction, which highlights the pathophysiological significance of β _1_-AA in liver fibrosis development.

## Supplementary Data

Supplementary Data is available at *Acta Biochimica et Biphysica Sinica* online.
